# Expression of MHC Class II Antigens During *Xenopus* Development

**DOI:** 10.1155/1990/67913

**Published:** 1990

**Authors:** Louis Du Pasquier, Martin F. Flajnik

**Affiliations:** 1 Basel Institute for Immunology Grenzacherstrasse 487 Basel CH-4058 Switzerland; 2 Department of Microbiology and Immunology University of Miami P.O. Box 01690 Miami Florida 33101 USA

## Abstract

Larval and adult forms of the amphibian Xenopus differ in their MHC class II .expression. In tadpoles, class II epitopes can be detected by monoclonal antibodies only on B cells, macrophages (whatever their location), spleen reticulum, thymus epithelium, and the pharyngobuccal cavity. In contrast, all adult T cells express class II on their surface. The transitions in class II expression occur at metamorphosis and are accompanied by other changes. The skin is invaded by class II positive dendritic cells, and the skin glands differentiate and also express class II. The gut, which expressed class II in discrete areas of the embryonic tissue, becomes invaded with B cells, and its epithelium also becomes class II positive.


*Developmental Immunology*, 1990, Vol. 1, pp. 85–95
Reprints available directly from the publisher
Photocopying permitted by license only

© 1990 Harwood Academic Publishers GmbH
Printed in the United Kingdom

### Expression of MHC Class II Antigens During *Xenopus* Development

LOUIS DU PASQUIER*† and MARTIN F. FLAJNIK‡

†*Basel Institute for Immunology, Grenzacherstrasse 487, CH-4058 Basel Switzerland*
‡*Department of Microbiology and Immunology, University of Miami, P.O. Box 01690, Miami, Florida, 33101*

Larval and adult forms of the amphibian *Xenopus* differ in their MHC class II expression. In tadpoles, class II epitopes can be detected by monoclonal antibodies only on B cells, macrophages (whatever their location), spleen reticulum, thymus epithelium, and the pharyngobuccal cavity. In contrast, all adult T cells express class II on their surface. The transitions in class II expression occur at metamorphosis and are accompanied by other changes. The skin is invaded by class II positive dendritic cells, and the skin glands differentiate and also express class II. The gut, which expressed class II in discrete areas of the embryonic tissue, becomes invaded with B cells, and its epithelium also becomes class II positive.

KEYWORDS: MHC class II antigens, amphibian development, tissue distribution.

### INTRODUCTION

During its ontogenetic development, the immune system of the anuran amphibian *Xenopus* clearly shows two different levels of organization and function corresponding to the larval and adult stages. Before metamorphosis, although the animals are immunologically competent, there is a paucity of thymus-dependent function, such as incomplete graft-rejection capacity (when compared to adults), little production of the most thymus-dependent Ig isotype IgY, and low graft versus host effector cell numbers. In addition, the antibody repertoire is different in larvae and adults (reviews in Flajnik et al., 1987; Du Pasquier et al., 1989). After metamorphosis, there exists a heightened T-cell function. The transition occurring at metamorphosis between these two systems is accompanied by a shift in the expression of MHC class I antigens. Not expressed on the surface of tadpole cells surface, MHC class I molecules appear during the climax of metamorphosis (Flajnik and Du Pasquier, 1988). Consistent with the fact that tadpoles are able to make antibody responses, the same class II molecules are present in both pre- and postmetamorphic stages, but little is known of their tissue distribution (Flajnik et al., 1986). Given the involvement of class II MHC molecules in the differentiation and function of the immune system (review in Carbone and Bevan, 1989), and now that several monoclonal antibodies against MHC epitopes are available (Flajnik et al., 1990), it seemed timely to investigate in detail the distribution and developmental aspects of these molecules in order to correlate functional aspects of the immune system with MHC antigen expression. The present work describes, with immunohistological and flow cytometry analyses, the expression of MHC class II molecules in *Xenopus* from early embryonic stages to sexual maturity.

*Corresponding author.

### RESULTS

The same class II molecules are expressed by tadpoles and adults, as detected by isoelectric focusing, at all developmental stages. The tissue distribution and the level of expression by T cells, however, are quite different during the larval and adult lives.

### Thymus

The thymus is the first organ to become lymphoid during development. It is also the first to become class II positive. The appearance of class II in this organ has been followed with more precision than others, given its importance in T-cell selection. Sections were taken twice a day during the early phase of development (i.e., the first week), and thus the sections contained the pharyngeal area before, during, and after the budding of the organ from pharyngeal endoderm.

The thymic epithelium does not express class II before its separation from the endoderm of the second pharyngeal pouch, which occurs at stage 46 of Nieuwkoop and Faber (1967) (approximately day 4.5 under our laboratory conditions). The following day, the organ becomes colonized by precursor cells present in the surrounding mesenchyme, which are strongly RC47$^+$ and therefore belong to the leukocyte lineage. Some of these large cells can be seen (Fig. 1) adhering to the outside part of the thymus. At this stage (5 days, stage 47), neither the thymus nor the RC47$^+$ cells are class II positive.

One to two day later (7 days, stage 48), the epithelium of the thymus gland becomes weakly class II positive. The staining increases progressively in intensity during the following week to reach the stable larval thymus staining pattern, which can be described as follows:

The cortical region stains more weakly than the medulla. The thymic epithelium and presumably

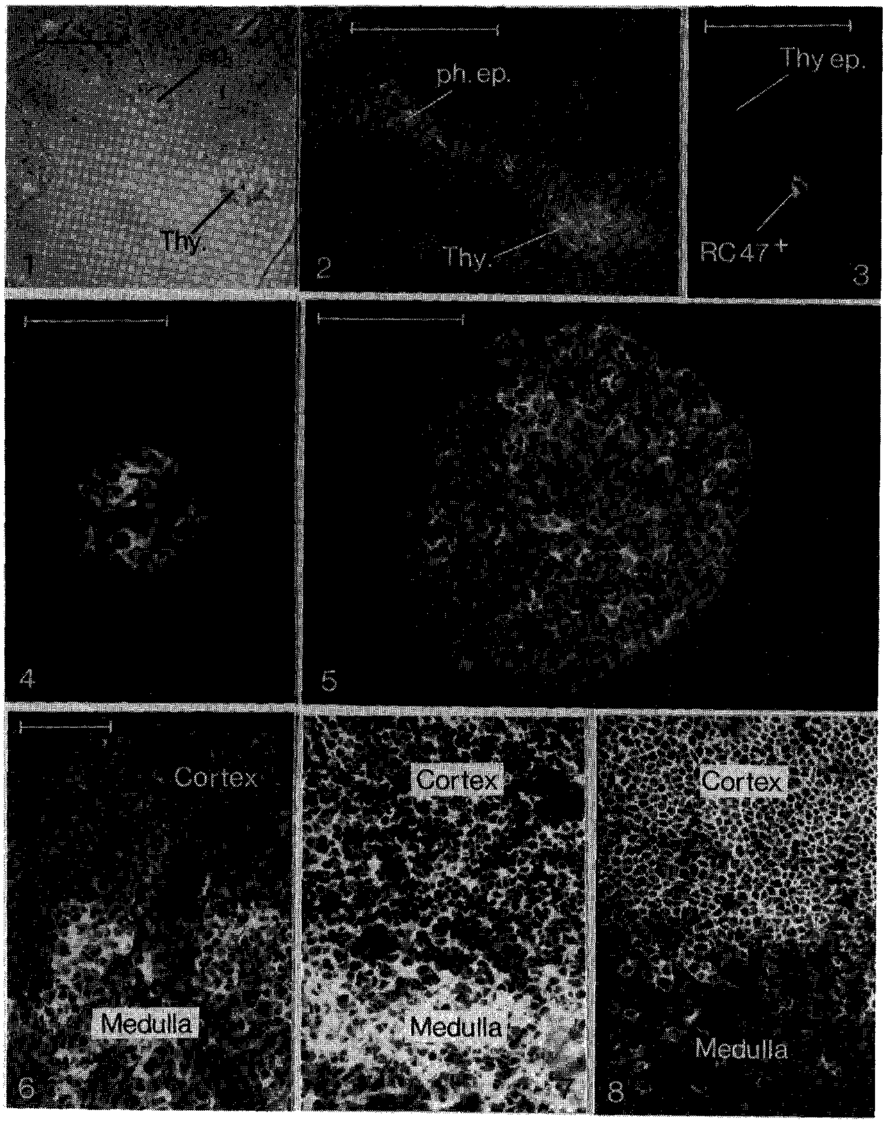

FIGURE 1. Ontogeny of class II expression in the thymus of Xenopus. (1) The thymus after separation from the pharyngeal pouch 5 days after fertilization. Phase contrast. ep.: epthelium. Thy.: thymus. (2) The budding thymus before separation from the pharyngeal pouch 4 days after fertilization does not stain with anticlass II reagents and is here shown stained with an antiubiquitous *Xenopus* antigen. Ph. ep.: pharyngeal epithelium. Thy.: thymus. (3) Cell of the hemopoietic lineage attached to the class II negative thymus anlage 5 days after fertilization (Thy. ep.), stained with antilymphocyte RC47 mAb (RC47$^+$). (4) Individualized thymus, 9 days after fertilization, stained with anticlass II mAb AM20. (5) Ibid., day 16. (6) Stage 56 thymus (ca. 40 days after fertilization). Medulla is strongly class II$^+$, whereas the cortex stains poorly AM20. (7) Young adult thymus with AM20. (8) Stage 58 thymus with AM22. The bar length is 100 microns.

antigen-presenting cells (APC) are apparently the only cells that express class II in the larval thymus, and they are more abundant in the medulla than in the cortex.

Single-cell immunofluorescence analysis of thymocytes demonstrated that larval thymocytes do not express class II (Fig. 1). This was confirmed by the absence of cortical staining on sections of larval thymus and further demonstrated by the observation that there was no cytoplasmic expression of class II molecules in cortical thymocytes. The weak staining seen in Fig. 1 is apparently due to epithelial cells.

During metamorphosis, the thymus is translocated toward the ear region (stages 60–65), and is deleted of at least 90% of its lymphocytes (Du Pasquier and Weiss, 1973). This depletion occurs mainly in the cortex, as shown by immunohistology. The entire thymus, which, at stage 65 consists almost exclusively of medulla, stains brightly with the class II specific mAbs. The use of MAb AM22, which stains all cortical thymocytes, confirms this observation, since it only stains a very thin layer of cortical lymphocytes at the maximum of thymic regression (stage 65) (Fig. 1 bis). For comparison, see the typical AM22 staining of larval thymus at stage 58 (Fig. 1). After completion of metamorphosis (stage 66), the thymus undergoes structural changes and a second histogenesis (Du Pasquier and Weiss, 1973; Clothier and Balls, 1985), and the general staining pattern reappears with distinct cortex, medulla, and their respective lymphocyte subpopulations.

The newly formed adult cortex, however, stains more brightly with the anticlass II reagents than the larval cortex did. This is because, unlike tadpoles, the adult cortical lymphocytes express the low levels of class II and are now recognized by the various anticlass II monoclonal antibodies (Figs. 1 and 2). The pattern of staining of adult thymocytes displays a continuous gradation of class II expression and apparently reflects a maturation process (Fig. 2), that is, the medullary thymocytes express more class II than the cortical thymocytes. The transition seems to occur sharply at the time of metamorphosis. It represents a major characteristic of the ontogeny of class II molecule expression, namely, that larval T cells do not express class II, but that adult T cells express easily detectable high levels of cell surface class II.

### Spleen

The spleen appears at stage 50, that is, about 15 days after hatching. At this stage, it consists of a few B-cell areas (two to three per organ) that can be identified with anti-Ig mAb staining; on serial sections, these B-cell areas are the most brightly stained with the class II specific mAbs (Fig. 3). Scattered cells, probably macrophages, are also visible; but there is no obvious staining of the T-cell areas, which become class II positive after metamorphosis (Fig. 3). The spleen lymphocytes have also been analyzed by FACS as single cells (Fig. 4). A large percentage of larval spleen lymphocytes do not express class II molecules, whereas in adult, apparently all lymphocytes are positive, FACS profiles of the splenic lymphocytes are consistent with the observation made with thymocytes, that adult T cells but not larval T cells express class II molecules. Figure 4 represents the FACS pattern of larval and adult splenocytes with various mAbs, which show (1) that B cells are more numerous in tadpoles than in adults, and (2) that about 50% of tadpole splenocytes are class II negative, whereas all adult splenic lymphocytes are positive. Double-staining analysis with antibody-conjugated fluorescent covaspheres

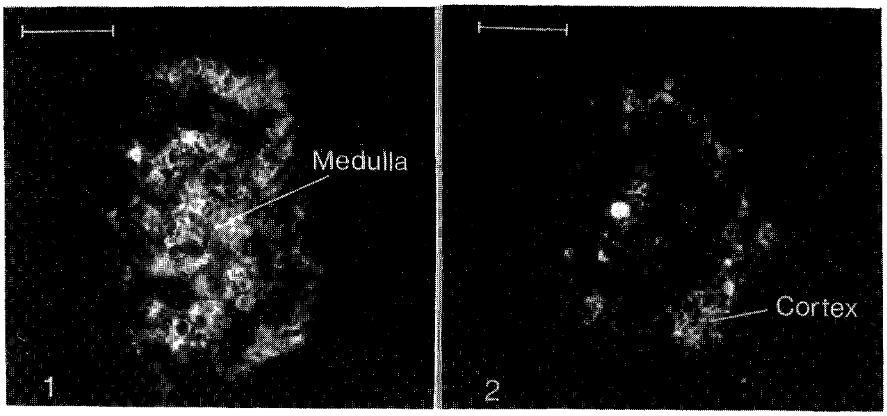

FIGURE 1 bis. Thymus at metamorphosis, stage 65. (1) Thymus section stained with class II-specific AM20 mAb. The monoclonal antibody stains practically all the surface of the entire section. The cortex is extremely reduced, as seen in (2), where the cortical lymphocyte-specific AM22 mAb stains only a discrete crown of cells. The bar length is 100 microns.

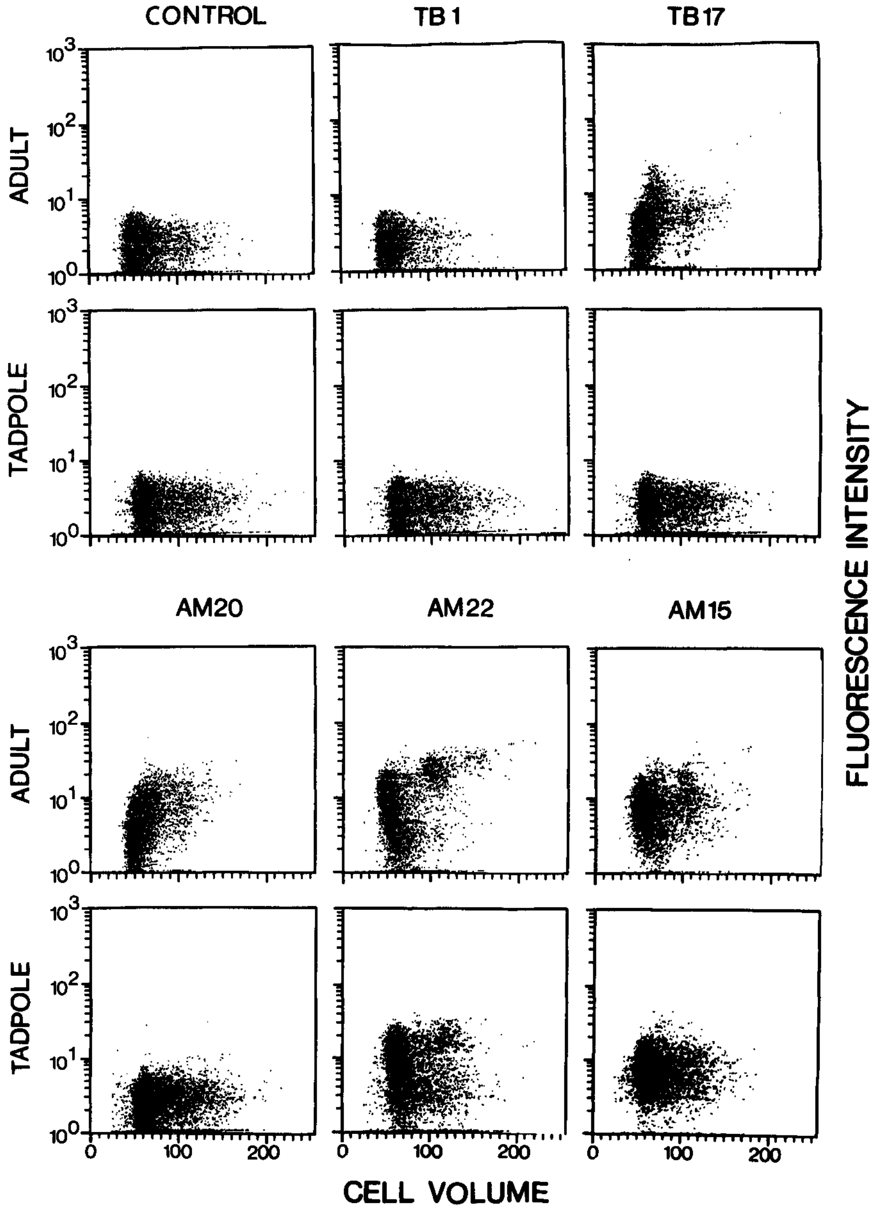

FIGURE 2. Tadpole thymocytes do not express cell-surface MHC molecules. FACS profiles of thymocytes from tadpoles and adults of the F strain "stained" with no antibody (control); TB1, specific for cytoplasmic class I molecules and negative on tadpole and adult cells; TB17, specific for class I, which is present only on adult cells; AM20, specific for class II, which is present only on adult cells; AM22 and AM15, specific for T cells, and both recognizing molecules on the surface of adult and tadpole cells. Coulter volume is on the X axis (linear scale) and fluorescence intensity is noted on a three-decade log scale on the Y axis.

(not shown) proved that the Ig-positive larval cells (B cells) are the major population of class II positive cells in the tadpole. The reticuloendothelium of the spleen, especially in the follicular areas, is class II positive. During larval life, the endothelium of the spleen seems to express higher amounts of class II molecules than the adult, but this is likely to be due to the absence of erythropoiesis in the larval spleen, which results in a tissue relatively richer in endothelium. Similarly, spleen from irradiated adult *Xenopus* (therefore, impaired in their erythropoiesis) show a pattern of class II expression in the endothelium similar to that of tadpoles (not shown).

### Liver

The hematopoietic layer of the liver, situated mainly under the organ's capsula, also contains many class $II^+$ cells, which correspond to the B cells identified with Ig-specific mAbs. Liver macrophages (equivalent to Küpffer's cells) are also clearly visualized with anticlass II mAb. There does not seem to be a

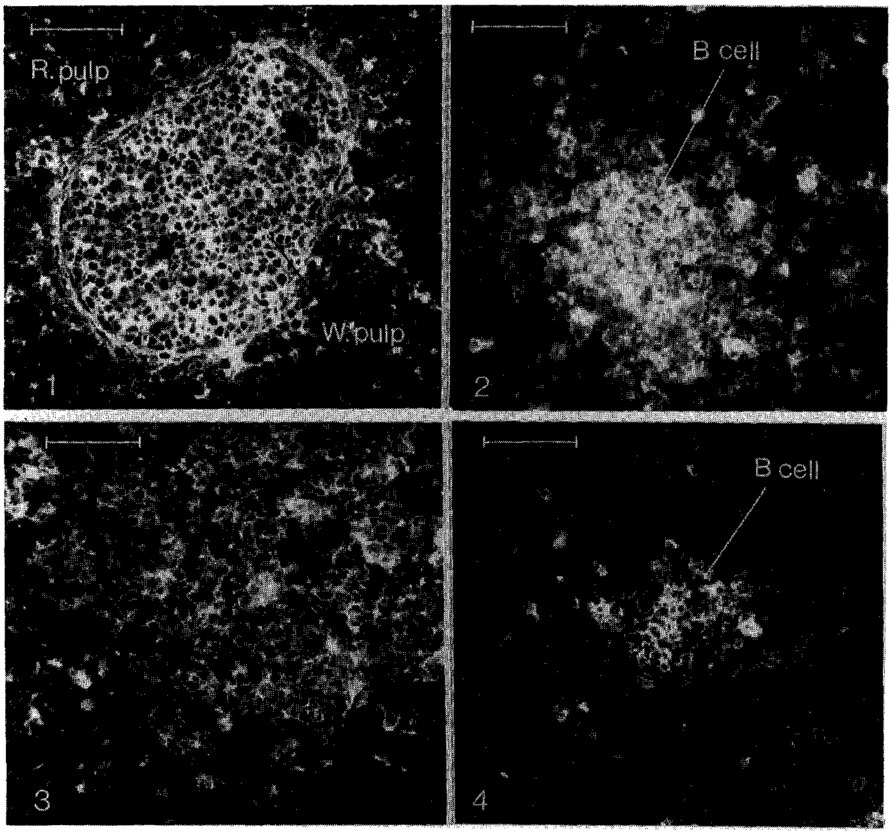

FIGURE 3. Ontogeny of class II expression in *Xenopus* spleen. (1) Adult spleen, stained with anticlass II AM20. Notice the scattered staining in the reticulum, corresponding mainly to T-cell staining, R. pulp: red pulp. W pulp: white pulp. (2) Adult spleen, a similar area B-cell focus in white pulp (B cell), stained with anti-IgM 10A9. (3) Tadpole spleen stage 54, anticlass II staining with AM20. Notice the abundant staining outside the B-cell focus corresponding to the spleen reticulum. The bar length is 100 microns. (4) Tadpole spleen stage 54, anti-Ig staining with 10A9, adjacent section. The bar length is 100 microns.

major ontogenetic difference between the larval and adult staining patterns of the liver (Fig. 5).

### Blood

Larval blood has not been analyzed with the cell sorter, and patterns of stains are shown only for the adult. They confirm the observation made in the spleen, that is, that all lymphocytes are stainable by the class II specific mAbs. Thrombocytes, granulocytes, and erythrocytes of *Xenopus laevis* do not express detectable amounts of class II molecules.

### Skin

Although the larval skin does not show staining with anticlass II during the first part of life, scattered, brightly stained cells can be detected from midlarval life onwards. As metamorphosis approaches, the dorsal skin of the head region starts to display large circular areas recognized by class II-specific mAbs (Fig. 5). We interpret these zones as the differentiating glands of the future skin, which in the adult are clearly class II positive. These glands stain more brightly at their apical extremity. The gland channels that permeate the skin epithelium are also class II positive at the membrane level (Fig. 5).

The skin of the adult *Xenopus* differs from the larval skin in many respects. Instead of a simple layer of epithelial cells over a layer of collagen, the adult skin consists of a multilayer epiderm and a derm characterized by the presence of many mucous and granular glands. No cells of the hematopoietic lineage are present in tadpole skin sections, or their frequency is so low that none can be detected. On the contrary, in the adult, the most inner part of the epiderm layer contains class II positive dendritic cells with long extensions at a frequency of about 1 cell per 20 epidermal cells. These cells, by their location, their functional aspects (they are more abundant in grafted tissues), and by their morphology are possibly homologous to the Langerhans cells of mammals (reviewed by Silberberg-Sinakin et al., 1980), and they may represent the skin APC of the adult frog.

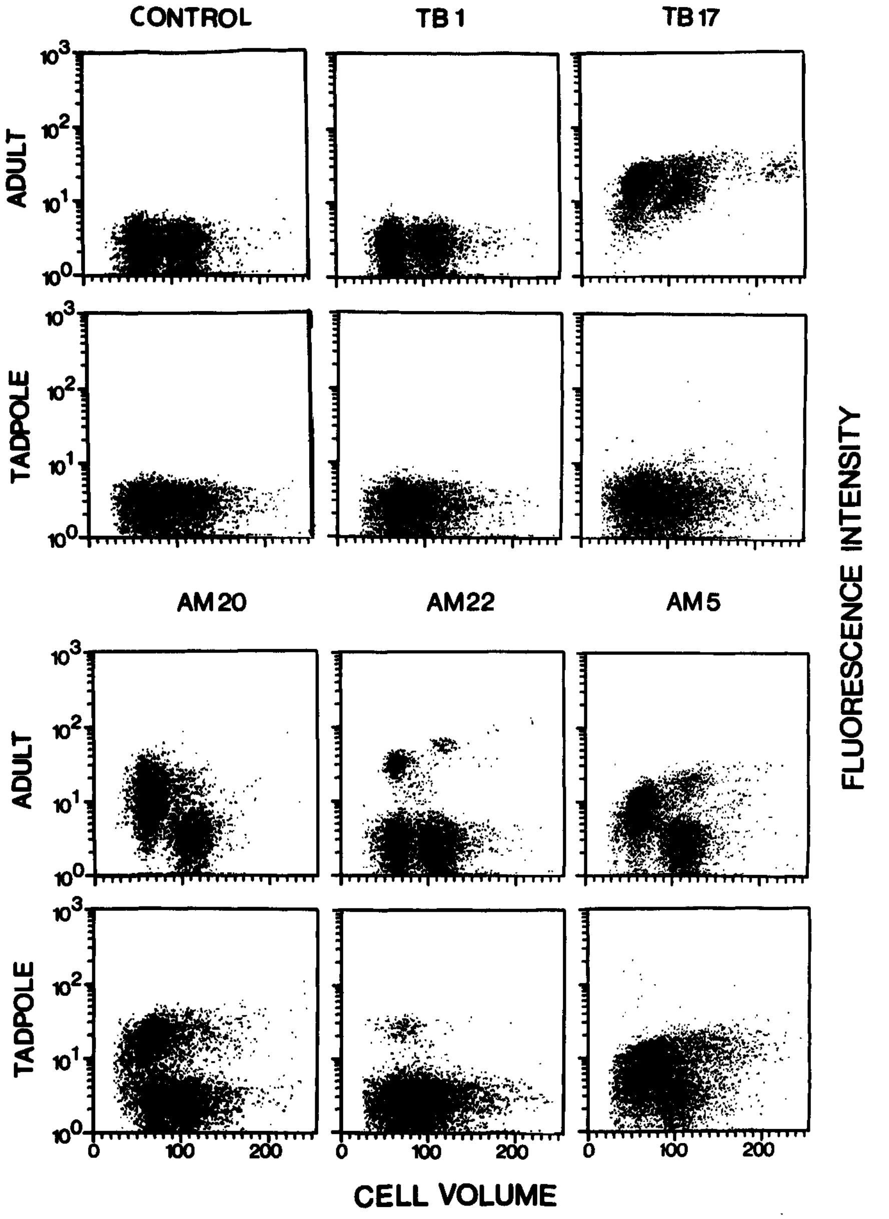

FIGURE 4. Mature lymphocytes show a restricted cell-surface distribution of MHC molecules. FACS profiles of splenocytes from tadpoles and adults of the F strain "stained" with the same antibodies as in the previous figure, except AM5, which recognizes all lymphocytes from tadpoles and adults. The erythrocytes are of larger volumes than the lymphocytes, which is conspicuous in adults, but not as apparent in tadpoles. The total number of lymphocytes in tadpoles can be seen by staining with the mAb AM5. Notice that tadpole splenocytes do not express class I molecules and only a subpopulation of the tadpole lymphocytes, in accordance with the number of B lymphocytes (not shown) express class II molecules.

### Pharnyx, Choanae, and Gut

The epithelium of the pharyngeal cavity and the choanae connected to the olfactory organs express class II molecules in larval life (Fig. 6). At the same time (stage 48), when the thymus becomes class II positive, some areas of the splanchnopleura around the larval gut also became class II positive. These areas contain hematopoietic cells, which can be stained by the RC47 mAb, and also by the apparently T-cell-specific mAb AM22. Moreover, an irregular pattern of class II expression can be detected with AM20 among many epithelial cells of the gut (Fig. 6). The adult gut is scattered with small nodules of class II positive B cells, and with plasma cells in the intestine epithelium. These cells also express class II. Moreover, the cells of the gut epithelium facing the lumen are selectively class II positive in their apical region (Fig. 6).

### Brain

Sections of the larval brain (Fig. 6) showed the presence of a loose layer of class II positive cells,

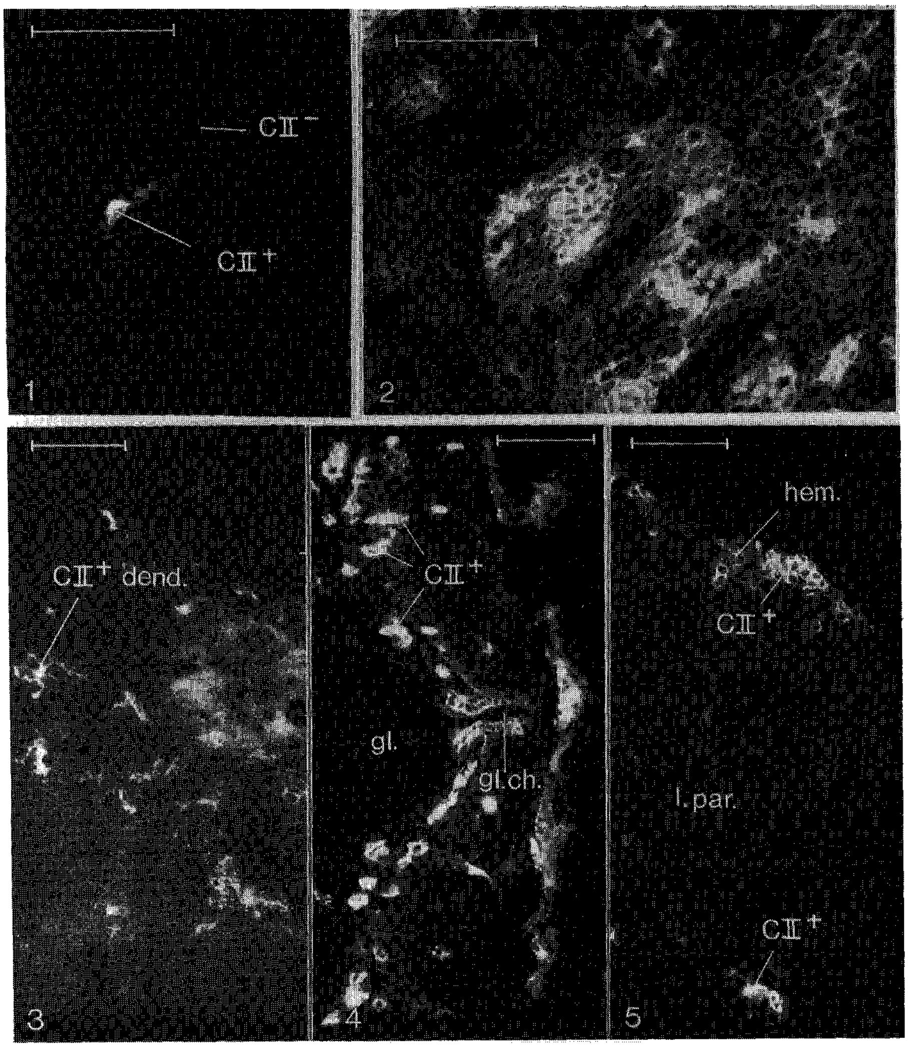

FIGURE 5. Ontogeny of class II expression in *Xenopus* skin and liver. (1) Islet of positive cells in tadpole skin, stage 54 AM20 anticlass II. (2) Tangential section of dorsal skin at metamorphosis. (3) Tangential section in adult skin showing the class II positive dendritic cells. (4) Transversal section in adult skin showing scattered as well as packed (cII$^+$) cells in the epiderm AM20 anticlass II, as well as one skin gland with its epidermal channel strongly class II positive gl. ch. (5) Tadpole liver stage 54, with the prepheric lymphoid cells and the inner macrophages staining brightly with anticlass II mAb AM20. l. par: liver parenchyme.

not interconnected near the walls of the ventricles. These cells may represent glial macrophages.

### DISCUSSION

The survey of MHC class II expression with monoclonal antibodies reinforces the hypothesis that the pre- and postmetamorphic immune systems are quite different. Class II expression, as judged by immunohistology, is at low levels during the first weeks of larval life. The intensity of staining of the cell surface of tissues during this time increases progressively and becomes stabilized at stages 51–52, which correlates with the onset of the larval immune competence. At metamorphosis, after class II expression of the larval type has been stable for 4 weeks, major changes occur within a week, such as migration of class II positive Langerhanslike cells into the skin (concurrent with the major changes in the anatomy of the skin), class II epitopes expression by the skin glands, and the appearance of class II on the surface of T cells. Therefore, the well-described stepwise acquisition of class II molecules during the ontogeny of mammals (Jenkinson et al., 1981; Natali et al., 1982) is not found for *Xenopus* due to the burst of new histogenesis at metamorphosis.

Nonlymphoid cell surface class II expression, especially in tadpoles, is concentrated in locations that are in direct contact with external stimuli. When class II molecules are found on epithelial cell surfaces in mammals, the expression occurs mainly in areas that contact with the internal environment.

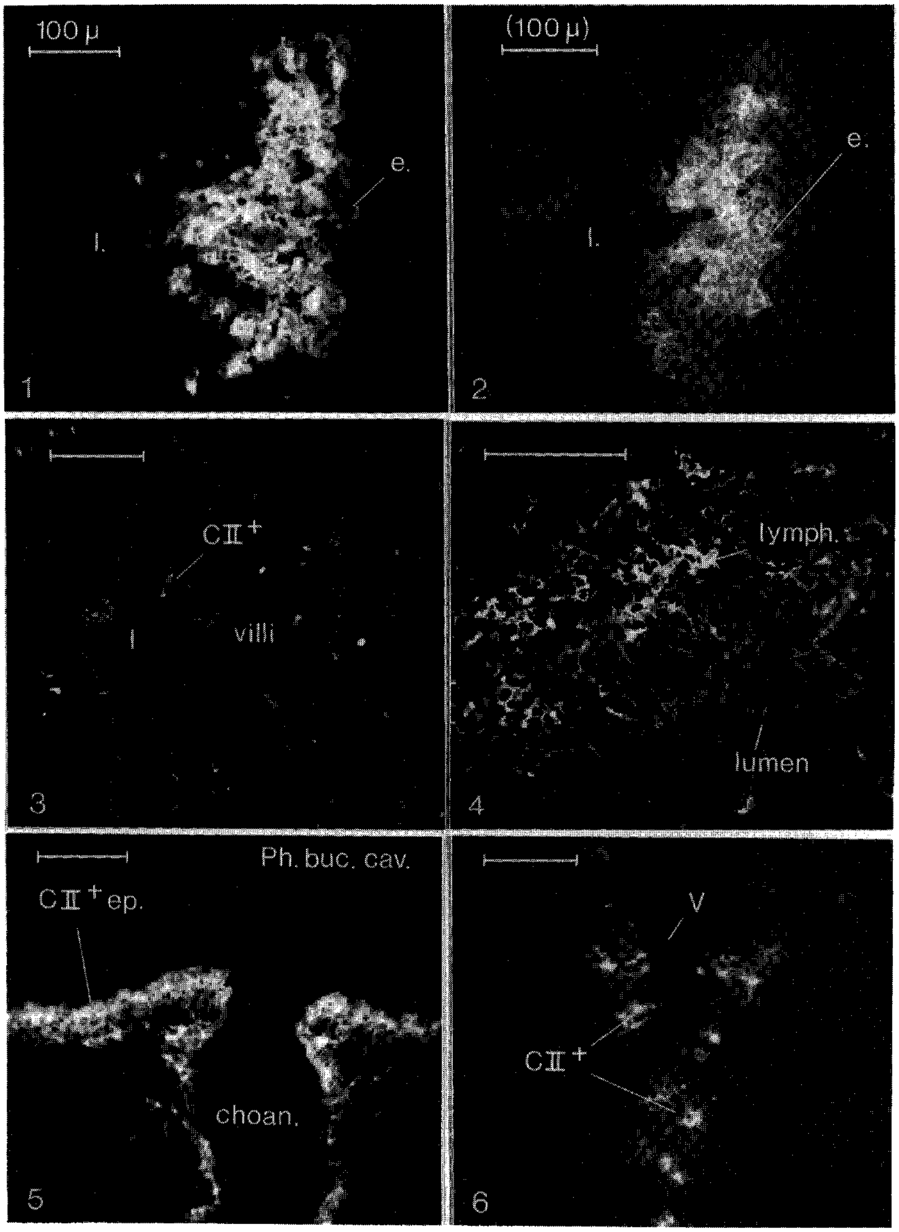

FIGURE 6. Larval and adult gut stained with anticlass II mAb AM20. (1) Larval gut stage 50, duodenum region stained with AM20. (2) Larval gut stage 50, duodenum region stained with AM22. l.: lumen. e.: epithelium. (3) In the epithelium with the stain (CII+) obvious in the part facing the lumen (l.). (4) In a region with lymphocyte accumulation, where the epithelium stains as well as the B lymphocytes (lymph.) (5) Pharyngo-buccal cavity in the choanes region, Ph. buc. cav. (choan). Epithelium strongly stained with AM20 (CII+ ep.). (6) Microglial cells in the brain, in the vicinity of the ventricule (V). Tadpole stage 58. The bar length is 100 microns.

In *Xenopus*, the situation is quite different; epithelium of the gill and pharyngobuccal cavities expose class II homogeneously on the surface of cells, including those regions in contact with the external environment (review in Klein, 1986), that is, facing the water or the contents of the digestive tract. This type of distribution suggests that class II molecules may be utilized for direct protection, either by allowing diverse cell types to present conventional antigen to T cells or by maintaining antigen in local concentrations, perhaps in a manner similar to the recent demonstrations of binding of native bacterial toxins to class II molecules (Fleischer, 1989). Epithelia that do not express class II, like the adult skin, contain dedicated APC (Langerhans cells). The class II negative gut epithelium of adults contains many plasma cells, showing that local antibody production is possible.

Tadpole skin epithelium is in general negative; however, a few islets of positive cells are apparent, which could correspond either to an area where local induction has taken place following infection due to the production of various stimulatory factors (e.g., interleukins or interferons) or to scattered areas of natural expression functioning as natural APC in the absence of dedicated APC. The class II expression by some gut areas in tadpoles further suggests that alternative presentation pathways may exist in tadpoles. Later in larval life, the gut is much poorer in class II positive areas. In adult life, however, class II expression can be detected in the intestine in the villi and in the apical regions of the cells facing the content of the digestive tract (Fig. 6). Thus, we suggest that at all times during ontogeny, and in most locations into the body, "units" of antigen-presenting function are present. The widespread distribution of class II in tadpoles may approximate the manner that antigen was presented in a more primitive vertebrate immune system.

Expression of MHC in the thymus during ontogeny shows that class II molecules are not involved in thymic budding nor for colonization by stem cells since these steps occur in the absence of class II expression. The environment for the education of T cells, however, is set up early in ontogeny, and is similar for the larval and adult thymus. The thymic epithelium, the first organ to become class II positive in ontogeny, retains such expression through metamorphosis. Thus, the frog fits in well with the known phylogeny of earliest class II expression, since the thymus, the first organ to become lymphoid, is also the first to become class II positive in almost all species that have been examined (Jenkinson et al., 1981; Natali et al., 1982; Pink and Gilmour, 1988). In absolute time comparisons with mammals and birds, *Xenopus*, due to its rapid immune system development, expresses class II molecules much earlier.

To some extent, the poor graft-rejection capacity and the suboptimal T–B collaboration during tadpole life must be related to the properties of larval T cells. There may simply be a paucity of larval T cells (Hsu and Du Pasquier, 1984a) or, rather, qualitative differences between adult and larval T cells. As previously proposed, in the absence of bona fide class I expression, the larval T-cell pool may be generated without selective amplification by positive selection of an entire subset of T cells. The consequences of the lack of class II expression on the larval T cells are more difficult to rationalize. In fact, the absence of class II on tadpole T cells may by more a consequence than a primary cause of the tadpole T-cell properties. The reason for the lack of class II expression could be due to the absence of a cytokine produced only in the adult thymus, which normally induces class I and class II on the maturing thymic lymphocytes. It has been shown, for example, that larval thymocytes, in contrast to adult thymocytes, cannot produce T-cell growth factors after lectin stimulation, but can nevertheless respond to such factors (Rollins-Smith et al., 1984; Cohen et al., 1987). Thus, either the thymocyte precursors are programmed differently during the larval and adult lives, or the thymic microenvironment induces different pathways of differentiation. If the latter possibility is true, it is known that different types of antigen-presenting cells colonize the thymus during its regeneration at metamorphosis and could contribute to the observed variations (Clothier and Balls, 1985). It is important to point out that the only differences in cell-surface molecule expression that we have so far observed is restricted to the MHC molecules; other T-cell/hematopoietic-cell-specific molecules are expressed in the same way during all stages of ontogeny.

Whether the changes in class II expression and the appearance of cell-surface class I molecules at the time of metamorphosis are under the control of the same factors will have to be elucidated later, at the biochemical (biosynthetic), endocrinological, and molecular genetic (promotor and promotor-binding factors) levels. It has recently been shown that the expression of class II molecules by adult T cells is dependent on the metamorphosis (Rollins-Smith and Blair, in press). Both for class I and class II, there is a sharp transition at the time of metamorphosis, and it is difficult not to hypothesize that the two types of expression might be linked. The apparent simultaneity of class II expression by T cells and of the ubiquitous class I expression on the cell surface suggests that these steps of MHC expression are under the same control pathways. This would allow, at the time of metamorphosis, a harmonious maturation of effector mechanisms in the new adult context. We have already proposed that MHC class I could serve as a restriction element involved in restructuring the organism at metamorphosis (Flajnik and Du Pasquier, 1990). If true, it is possible that those tissues that coexpress class I and class II at metamorphosis are most susceptible to postulated cells of the immune system involved in restructuring the organism.

### MATERIALS AND METHODS

### Animals

*Xenopus laevis* of the F strain (*f* haplotype) and (*Xenopus laevis*) ×(*X. gilli*) hybrids ($LG_{15}$) *a/c* haplotypes have been used throughout all experiments. Their production has been detailed elsewhere (Kobel and Du Pasquier, 1975).

Embryos and larvae were frozen in toto on dry ice and embedded in OCT before sectioing with a Leitz Cryostat microtome. For adult, organs were dissected out (skin, thymus, spleen, gut, liver, brain, etc.) and frozen before being sectioned.

### Sections

Tissue sections, 5$\mu$, were cut at −18°C and recovered on multilodge slides (Hölzel) and fixed for 30 s in acetone. They were then incubated in a humidified chamber with the first reagent (monoclonal antibodies) for 12–15 h at 4°C. After washing with 2% BSA in PBS containing 0.05% Tween 20, the next incubation was started with goat antimouse Ig labeled with fluorescein isothiocynate (Nordic), absorbed on *Xenopus* erythrocytes (10 volumes of reagent and 1 volume of pelleted *Xenopus* red cells 0°C, 0.5 h). After 1 h at room temperature, the slides were washed with PBS and mounted on PBS and 25 mg/ml 1–4 Diazabicyclo (2.2.2) octan (Fluka) (DABCO) and 20% glycerol. DABCO prevents the fluorescence from bleaching. Sections were observed with a Zeiss microscope using 16×, 25×, and 50×water immersion objectives (Du Pasquier et al., 1985).

### Monoclonal Antibodies

Four anti-*Xenopus* class II mAbs, all showing good reactivity with F and $LG_{15}$, have been used: 14A2, AM20, 1C9, and 3E8. Their productions and properties have been described elsewhere (Flajnik et al., 1990).

Several other mAbs were used as controls. These included $TB_{17}$ (anti-MHC class I recognizing cell surface and cytoplasmic class I molecules), RC47 (specific for cells of the leukocyte lineage from very early stages of development), AM5, AM15, AM22 (specific for subpopulations of lymphocytes), and anti-Ig$\mu$ mAb 10A9 (specific for $\mu$) (Hsu and Du Pasquier, 1984b).

### Cell Analyzer

For single-cell immunofluorescence analysis, the fluorescence cell analyzer (Becton Dickinson) was used on suspensions of spleen, blood, or thymus cells, according to a protocol already described (Flajnik and Du Pasquier, 1988).

### ACKNOWLEDGMENTS

We thank Michaela Manes and Birgit Kugelberg for expert technical assistance, Dr. Joseph Schwager for critical reading of the manuscript, and Janette Millar for typing the manuscript. The Basel Institute for Immunology, Grenzacherstrasse 487, CH-4058 Basel, Switzerland.

*(Received April 25, 1990)*

*(Accepted May 3, 1990)*

### REFERENCES

Carbone F.R., and Bevan M.J. (1989). Major histocompatibility complex control of T cell recognition. *In:* Fundamental Immunology. Paul W. E., ed. (New York: Raven Press), pp. 541–567.

Clothier R.H., and Balls, M. (1985). Structural changes in the thymus glands of *Xenopus laevis* during development. *In:* Metamorphosis. Balls M., and Bownes M., Eds. (Oxford: Clarendon Press), pp. 332–359.

Cohen N., Watkins D., and Parsons S.C.V. (1987). Interleukins and T-cell ontogeny in *Xenopus laevis*. *In:* Developmental and Comparative Immunology. Cooper E.L., Langlet C., and Bierne T., Eds. (New York: Alan R. Liss Inc.), pp. 53–68.

Du Pasquier L., Flajnik M.F., Guiet C., and Hsu E. (1985). Methods used to study the immune system of *Xenopus* (Amphibia, Anuran). *In:* Immunological Methods III. Lefkovits I., and Pernis B., Eds. (Orlando, FL: Academic Press), pp. 425–465.

Du Pasquier L., Schwager J., and Flajnik M.F. (1989). The immune system of the *Xenopus*. Ann. Rev. Immunol. **7:** 251–275.

Du Pasquier L., and Weiss N. (1973). The thymus during the ontogeny of the toad *Xenopus laevis:* Growth, membrane-bound immunoglobulins and mixed lymphocyte reaction. Eur J. Immunol. **3:** 773.

Flajnik M.F., and Du Pasquier L. (1988). MHC class I antigens as surface markers of adult erythrocytes during the metamorphosis of *Xenopus*. Dev. Biol. **128:** 198–206.

Flajnik M.F., and Du Pasquier L. (1990). Changes in the expression of the major histocompatibility complex during the metamorphosis of *Xenopus*. *In:* Developmental Biology, UCLA Symposium on Molecular and Cellular Biology, New Series, vol. 125, Davidson E., Rudeman I., and Posokeny J., Eds. (New York: Wiley Liss Inc.), pp. 215–224.

Flajnik M.F., Ferrone S., Cohen N., and Du Pasquier L. (1990). Evolution of the MHC: Antigenicity and unusual tissue distribution of *Xenopus* (frog) class II molecules. Mol. Immunol. **27:** 451–462.

Flajnik M.F., Horan P.K., and Cohen N. (1983). A flow cytometric analysis of the embryonic origin of lymphocytes in diploid/triploid chimeras *Xenopus laevis*. Dev. Biol. **104:** 247–254.

Flajnik M.F., Hsu E., Kaufman J.F., and Du Pasquier L. (1987). Changes in the immune system during metamorphosis of *Xenopus*. Immunol. Today **8**: 58–64.

Flajnik M.F., Kaufman J.F., Hsu E., Manes M., Parisot R., and Du Pasquier L. (1986). Major histocompatibility complex-encoded class I molecules are absent in immunologically competent *Xenopus* before metamorphosis. J. Immunol. **137**: 3891–3899.

Fleischer B. (1989). Bacterial toxins as probes for the T cell antigen receptor. Immunol. Today **10**: 262–264.

Hsu E., and Du Pasquier L. (1984a). Ontogeny of the immune system in *Xenopus* II. Antibody repertoire differences between larvae and adults. Differentiation **28**: 116–122.

Hsu E., and Du Pasquier L. (1984b). Studies on *Xenopus* immunoglobulins using monoclonal antibodies. *Mol. Immunol.* **21**: 257.

Jenkinson E.J., van Ewjik W., and Owen J.J.T. (1981). Major histocompatibility complex antigen expressions on the epithelium of the developing thymus in normal and nude mice. J. Exp. Med. **153**: 280.

Klein J. (1986). Natural history of the major histocompatibility complex (New York: John Wiley).

Kobel H.R., and Du Pasquier L. (1975). Production of large clones of histocompatible fully identical clawed toads (*Xenopus*). Immunogenetics **12**: 546.

Natali P.G., Russo C., Nig A.K., Nicotra M.R., Apollonj C., Pellegrino M.A., and Ferrone S. (1982). Ontogeny of human Ia antigens. Cell. Immunol. **73**: 385.

Nieuwkoop P.D., and Faber J. (1967). Normal table of *Xenopus laevis*. (Amsterdam: North Holland).

Pink J.R.L., and Gilmour D.G. (1988). Surface antigens of avian blood cells *In:* Differentiation Antigens in Lymphopoietic Tissues. Miyasaka M., and Trnka Z., (New York: Marcel Dekker), pp. 361–385.

Rollins-Smith L., and Blair P. (1990). The expression of class II major histocompatibility complex antigens on adult T cells in *Xenopus* is metamorphosis dependent. Dev. Immunol. **1**: 97–104.

Rollins-Smith L.A., Parsons S.C.V., and Cohen N. (1984). During frog ontogeny, PHA and ConA responsiveness of splenocytes precedes that of thymocytes. Immunology **52**: 491–500.

Silberberg-Sinakin I., Gigli I., Baer R.L., and Thorbecke G.J. (1980). Langerhans cells: Role in contact hypersensitivity and relationship to lymphoid dendritic cells and to macrophages. Immunol. Rev. **53**: 203–232.

